# Taste compounds, affecting factors, and methods used to evaluate chicken soup: A review

**DOI:** 10.1002/fsn3.2501

**Published:** 2021-08-10

**Authors:** Lili Zhang, Zhilin Hao, Chao Zhao, Yuyu Zhang, Jian Li, Baoguo Sun, Yizhuang Tang, Meixiang Yao

**Affiliations:** ^1^ College of Food Science and Engineering Tianjin University of Science and Technology Tianjin China; ^2^ Beijing Key Laboratory of Flavor Chemistry Beijing Technology and Business University Beijing China; ^3^ College of Food Science Fujian Agriculture and Forestry University Fujian China; ^4^ Jiangxi Jiangzhong Diet Therapy Technology Co., Ltd Jiujiang China

**Keywords:** affecting factors, chicken soup, evaluation methods, taste compounds

## Abstract

The taste of chicken soup is dependent upon various taste substances and human senses. More than 300 nonvolatile compounds reportedly exist in chicken/chicken soup. The primary purpose of this review was to elaborate on the prominent taste substances, the taste evaluation methods, and the factors affecting the taste of chicken soup. Most taste‐active compounds with taste descriptions and thresholds in chicken soup were summarized. The application of sensory evaluation, liquid chromatography, electronic tongue, and other evaluation methods in chicken soup taste analysis were elaborated. The effects of genetic constitution, preslaughter, processing, and storage on chicken soup taste had been discussed. Nucleotides (especially inosine 5′‐monophosphate), amino acids and their derivatives, organic acids, sugars, and peptides play a vital role in the taste attributes of chicken soup. Combining of liquid chromatography and mass spectrometry enables qualitative and quantitative analysis of taste‐active compounds in chicken soup, aiding the exploration of key taste‐active compounds. The electronic tongue application helps the overall taste perception of the soluble taste‐active compounds present in chicken soup samples. Postmortem aging and stewing for a prolonged duration are effective techniques for improving the taste quality of chicken soup. The washing of preprocessing, the cooking temperature of processing, and the storage conditions also exert a significant impact on the taste of chicken soup.

## Graphical abstract

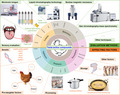

## 1 INTRODUCTION

The chicken products, featured with their high protein content, low‐fat content, and affordable price, gain popularity widely in China. As per findings reported by a previous study, 100 g of chicken contained 167.0 kcal energy, 19.3 g protein, 9.4 g fat, and 69.0 g water (chicken’s nutrient content from “China Food Composition Tables Standard Edition (Yang, 2018)”). Chicken is rich in various vitamins and is a reliable source of phosphorus, copper, iron, zinc, and other minerals. Compared to beef and pork, chicken fat contains more unsaturated fatty acids (oleic acid and linoleic acid), and these fatty acids play an important role in the formulation of strategies for the treatment and prevention of cardiovascular diseases (Chiu et al., 2007). There is no religious restriction on the consumption of chicken, and it has the characteristics of low price, convenient cooking, and timely supply of processed products (Fu et al., 2006). Available data indicate that the proportion of chicken consumption in China has shown a steady growth trend in recent years (https://downloads.usda.library.cornell.edu/usda‐esmis/files/73666448x/t435h4995/vt1519922/livestock_poultry.pdf [the United States Department of Agriculture – USDA]). The reason is not only the substitution of poultry for pork under the influence of African swine fever, but also that consumers prefer to choose poultry for their health (Woźniak et al., 2016).

Stew is one of the popular ways to cook chicken in China. As a result, chicken soup has become an indispensable part of several meals (Kurobayashi et al., 2008). As the taste of chicken soup remarkably affects its palatability and consumer acceptance, it is one of the main characteristic factors of chicken soup (Drewnowski & Darmon, 2005). Considering consumers’ pursuit of delicacy, any subtle changes in chicken soup taste can considerably affect consumer choice and purchase. Therefore, evaluation and research on the taste of chicken soup are of significance for promoting the industrialization of high‐quality chicken soup. Over the past half‐century, several researchers have engaged efforts for the improvement of characteristic taste‐active compounds of chicken soup to facilitate its industrial production. However, the complex interactions between various flavor compounds, coupled with multiple influencing factors during breeding and processing, pose challenges to maintain of consistency in the sensory quality of chicken soup. In this article, the taste‐active compounds contributing to the taste of chicken soup, the factors affecting chicken soup taste, and the methods used to evaluate the taste of chicken soup were discussed.

## 2 TASTE‐ACTIVE COMPOUNDS IN CHICKEN SOUP

Identification of characteristic taste‐active compounds in chicken soup is essential for the analysis of chicken soup taste. Thus far, more than 300 small molecular compounds have been reported in chicken/chicken soup (Table 1), of which 91 are taste‐active compounds (Jayasena et al., 2013a; Pippen et al., 1960; Wang et al., 2020; Yang et al., 2018; Zhang, Zhao, et al., 2019). Identifying the key flavor components in chicken soup remains difficult when studying the flavor characteristics of chicken soup due to the presence of a large number of taste‐active components in the soup. The characteristic taste of chicken soup could be attributed to the taste‐active compounds in chicken as well as those generated during stewing. As a complex attribute, the taste of chicken soup is determined by the comprehensive analysis of several different taste‐active compounds rather than one or two taste‐active compounds.

**TABLE 1:** Small molecular compounds have been reported in chicken/chicken soup

No.	Compound^a^	Reference
1	Methionine	You et al. (2019)
2	Valine	You et al. (2019)
3	Leucine	You et al. (2019)
4	Phenylalanine	You et al. (2019)
5	Isoleucine	You et al. (2019)
6	Threonine	You et al. (2019)
7	Lysine	You et al. (2019)
8	Glutamic acid	You et al. (2019)
9	Aspartic acid	You et al. (2019)
10	Glycine	You et al. (2019)
11	Serine	You et al. (2019)
12	Alanine	You et al. (2019)
13	Histidine	You et al. (2019)
14	Arginine	You et al. (2019)
15	Proline	Dunkel and Hofmann (2009)
16	Cystine	You et al. (2019)
17	Tyrosine	Zhang et al. (2020)
18	Cysteine	Zhan et al. (2020)
19	Ammonium chloride	Zhang et al. (2020)
20	DL‐O‐Phosphoserine	Zhang et al. (2020)
21	Taurine	Zhang et al. (2020)
22	Urea	Zhang et al. (2020)
23	Sarcosine	Zhang et al. (2020)
24	Cystathionine	Zhang et al. (2020)
25	β‐Alanine	Zhang et al. (2020)
26	4‐Aminobutyric acid	Zhang et al. (2020)
27	Ethanolamine	Zhang et al. (2020)
28	5‐Hydroxy‐DL‐Lysine	Zhang et al. (2020)
29	L(+)‐Ornithine	Zhang et al. (2020)
30	L‐Glutamine	Dunkel and Hofmann (2009)
31	L‐asparagine	Dunkel and Hofmann (2009)
32	Hydroxyproline	Miyaki et al. (2015)
33	α‐Aminoadipic acid	Pérez‐Palacios et al. (2017)
34	Allo‐isoleucine	Pérez‐Palacios et al. (2017)
35	β‐Aminoisobutyric acid	Pérez‐Palacios et al. (2017)
36	N,N‐Dimethylglycine	Xiao, Ge, et al. (2019)
37	Phosphoserine	Aliani and Farmer (2005)
38	Oxalic acid	Zhang et al. (2020)
39	Tartaric acid	Zhang et al. (2020)
40	Formic acid	Zhang et al. (2020)
41	Lactic acid	Zhang et al. (2020)
42	Acetic acid	Zhang et al. (2020)
43	Pyroglutamic acid	Zhang et al. (2020)
44	Citric acid	Zhang et al. (2020)
45	Fumaric acid	Zhang et al. (2020)
46	Succinic acid	Zhang et al. (2020)
47	Malic acid	Dunkel and Hofmann (2009)
48	C4:0	Xiao, Luo, et al. (2019)
49	C6:0	Xiao, Luo, et al. (2019)
50	C8:0	Xiao, Luo, et al. (2019)
51	C10:0	Xiao, Luo, et al. (2019)
52	C11:0	Xiao, Luo, et al. (2019)
53	C12:0	Zhang, Zhao, et al. (2019)
54	C13:0	Xiao, Luo, et al. (2019)
55	C14:0	Yang et al. (2018)
56	C15:0	Xiao, Luo, et al. (2019)
57	C16:0	Yang et al. (2018)
58	C17:0	Zhang, Zhao, et al. (2019)
59	C18:0	Yang et al. (2018)
60	C21:0	Zhang, Zhao, et al. (2019)
61	C22:0	Yang et al. (2018)
62	C24:0	Yang et al. (2018)
63	C14:1	Zhang, Zhao, et al. (2019)
64	C16:1	Yang et al. (2018)
65	C17:1	Rikimaru and Takahashi (2010)
66	C18:1 n‐9t	Zhang, Zhao, et al. (2019)
67	C18:1 n‐9c	Zhang, Zhao, et al. (2019)
68	C18:2	Yang et al. (2018)
69	C18:3	Yang et al. (2018)
70	C20:1	Zhang, Zhao, et al. (2019)
71	C20:2	Zhang, Zhao, et al. (2019)
72	C20:3	Yang et al. (2018)
73	C20:4	Zhang, Zhao, et al. (2019)
74	C20:5	Yang et al. (2018)
75	C22:1	Zhang, Zhao, et al. (2019)
76	C22:4	Yang et al. (2018)
77	C22:5	Yang et al. (2018)
78	C22:6	Yang et al. (2018)
79	C24:1	Yang et al. (2018)
80	5′‐UMP	Dunkel and Hofmann (2009)
81	5′‐CMP	Zhang et al. (2020)
82	5′‐GMP	You et al. (2019)
83	5′‐AMP	Zhang et al. (2020)
84	5′‐IMP	Fukuuchi et al. (2018)
85	3′:5′‐cAMP	Dunkel and Hofmann (2009)
86	ATP	Fukuuchi et al. (2018)
87	ADP	Fukuuchi et al. (2018)
88	AMP	Fukuuchi et al. (2018)
89	Adenosine	Fukuuchi et al. (2018)
90	Adenine	Fukuuchi et al. (2018)
91	GTP	Fukuuchi et al. (2018)
92	GDP	Fukuuchi et al. (2018)
93	GMP	Fukuuchi et al. (2018)
94	XMP	Fukuuchi et al. (2018)
95	Guanosine	Fukuuchi et al. (2018)
96	Guanine	Fukuuchi et al. (2018)
97	Inosine	Fukuuchi et al. (2018)
98	Hypoxanthine	Fukuuchi et al. (2018)
99	Xanthosine	Fukuuchi et al. (2018)
100	Xanthine	Fukuuchi et al. (2018)
101	Uridine	Xiao, Ge, et al. (2019)
102	Glucose	Dunkel and Hofmann (2009)
103	Fructose	Dunkel and Hofmann (2009)
104	Sucrose	Dunkel and Hofmann (2009)
105	Inositol	Dunkel and Hofmann (2009)
106	Xylitol	Dunkel and Hofmann (2009)
107	Ethylene glycol	Dunkel and Hofmann (2009)
108	Ribitol	Dunkel and Hofmann (2009)
109	Glucose‐1‐phosphate	Xiao, Tahara, et al. (2019)
110	Ribose phosphate	Aliani and Farmer (2002)
111	Glucose phosphate	Aliani and Farmer (2002)
112	Ribose‐5‐phosphate	Aliani and Farmer (2002)
113	Ribose	Yang et al. (2018)
114	2‐Aminobutyrate	Xiao, Ge, et al. (2019)
115	Butyrate	Xiao, Ge, et al. (2019)
116	2‐Hydroxybutyrate	Xiao, Ge, et al. (2019)
117	4‐Hydroxybutyrate	Xiao, Ge, et al. (2019)
118	Pyruvate	Xiao, Ge, et al. (2019)
119	Chloride	Dunkel and Hofmann (2009)
120	Phosphate	Dunkel and Hofmann (2009)
121	K_2_HPO_4_	Nishimura et al. (2016)
122	MgCl_2_	Nishimura et al. (2016)
123	CaCl_2_	Nishimura et al. (2016)
124	${\text{PO}}_{4}^{3‐}$	Qi et al. (2017)
125	^23^Na	Choi (2011)
126	^24^Mg	Choi (2011)
127	^27^Al	Mi et al. (2018)
128	^31^P	Choi (2011)
129	^39^K	Choi (2011)
130	^43^Ca	Mi et al. (2018)
131	^44^Ca	Mi et al. (2018)
132	^45^Sc	Mi et al. (2018)
133	^51^V	Mi et al. (2018)
134	^52^Cr	Mi et al. (2018)
135	^55^Mn	Mi et al. (2018)
136	^56^Fe	Mi et al. (2018)
137	^59^Co	Mi et al. (2018)
138	^60^Ni	Mi et al. (2018)
139	^63^Cu	Mi et al. (2018)
140	^66^Zn	Mi et al. (2018)
141	^75^As	Mi et al. (2018)
142	^78^Se	Mi et al. (2018)
143	^85^Rb	Mi et al. (2018)
144	^88^Sr	Mi et al. (2018)
145	^89^Y	Mi et al. (2018)
146	^95^Mo	Mi et al. (2018)
147	^101^Ru	Mi et al. (2018)
148	^107^Ag	Mi et al. (2018)
149	^111^Cd	Mi et al. (2018)
150	^125^Te	Mi et al. (2018)
151	^133^Cs	Mi et al. (2018)
152	^137^Ba	Mi et al. (2018)
153	^139^La	Mi et al. (2018)
154	^140^Ce	Mi et al. (2018)
155	^141^Pr	Mi et al. (2018)
156	^146^Nd	Mi et al. (2018)
157	^157^Gd	Mi et al. (2018)
158	^175^Lu	Mi et al. (2018)
159	^178^Hf	Mi et al. (2018)
160	^193^Ir	Mi et al. (2018)
161	^195^Pt	Mi et al. (2018)
162	^197^Au	Mi et al. (2018)
163	^205^Tl	Mi et al. (2018)
164	^208^Pb	Mi et al. (2018)
165	^232^Th	Mi et al. (2018)
166	^119^Sn	Das and Das (1989)
167	Ammonium	Dunkel and Hofmann (2009)
168	1‐Methyl‐L‐histidine	Zhang et al. (2020)
169	3‐Methyl‐L‐histidine	Zhang et al. (2020)
170	Anserine	Zhang et al. (2020)
171	Carnosine	Zhang et al. (2020)
172	Glutathione	Xiao, Tahara, et al. (2019)
173	β‐Alanyl‐N‐methyl‐L‐histidine	Dunkel and Hofmann (2009)
174	β‐Alanyl‐L‐histidine	Dunkel and Hofmann (2009)
175	β‐Alanylglycine	Dunkel and Hofmann (2009)
176	Pro‐Hyp	Kouguchi et al. (2012)
177	Hyp‐Gly	Kouguchi et al. (2012)
178	LVQY	Wang et al. (2020)
179	VHAHS	Wang et al. (2020)
180	AQNSYPHA	Wang et al. (2020)
181	AEQYRLVG	Wang et al. (2020)
182	WVNEEDHL	Zhang, Ma, et al. (2019)
183	NSLEGEFKG	Zhang, Ma, et al. (2019)
184	KDLFDPVIQD	Zhang, Ma, et al. (2019)
185	AD	Kong et al. (2017)
186	AM	Kong et al. (2017)
187	HS	Kong et al. (2017)
188	VE	Kong et al. (2017)
189	AE	Kong et al. (2017)
190	DAG	Kong et al. (2017)
191	ED	Kong et al. (2017)
192	AEA	Kong et al. (2017)
193	VT	Kong et al. (2017)
194	AH	Kong et al. (2017)
195	AF	Kong et al. (2017)
196	TE	Kong et al. (2017)
197	DA	Maehashi et al. (1999)
198	DV	Maehashi et al. (1999)
199	EE	Maehashi et al. (1999)
200	EV	Maehashi et al. (1999)
201	ADE	Maehashi et al. (1999)
202	DEE	Maehashi et al. (1999)
203	DES	Maehashi et al. (1999)
204	EEN	Maehashi et al. (1999)
205	SPE	Maehashi et al. (1999)
206	EPAD	Maehashi et al. (1999)
207	LRGDVGPVRTGEQG	Lin et al. (2016)
208	IGTGVSGGEEGALKGPS	Lin et al. (2016)
209	LGAGEKGPVGY	Lin et al. (2016)
210	IPPGGHYGEDAHGY	Lin et al. (2016)
211	EPPPVKVPEEPK	Lin et al. (2016)
212	RGDPGPVGPVGPA	Lin et al. (2016)
213	LPIKDPHVDSA	Lin et al. (2016)
214	FDAAKSPTGQ	Lin et al. (2016)
215	VLSAADKNNVKG	Lin et al. (2016)
216	EPPPPKEPEVPKK	Lin et al. (2016)
217	HYAQDSGVAGAPPN	Lin et al. (2016)
218	PAPPPEEKPRIK	Lin et al. (2016)
219	SLPKDTPGFQH	Lin et al. (2016)
220	TEGGETLTVK	Lin et al. (2016)
221	GEAAPYLRKS	Lin et al. (2016)
222	GFAGDDAPRA	Lin et al. (2016)
223	VFVVHPKES	Lin et al. (2016)
224	YDTPDMVRA	Lin et al. (2016)
225	TPPKIDSPRA	Lin et al. (2016)
226	PAEDNIQSRS	Lin et al. (2016)
227	TESGETLTVK	Lin et al. (2016)
228	VPVPVSRK	Lin et al. (2016)
229	GEAAPYLRK	Lin et al. (2016)
230	LPPKRPP	Lin et al. (2016)
231	VGNEYVTK	Lin et al. (2016)
232	DMIPAQK	Lin et al. (2016)
233	AHDGGRYY	Lin et al. (2016)
234	TPPPMQAK	Lin et al. (2016)
235	VVHPKESF	Lin et al. (2016)
236	YEAFVKH	Lin et al. (2016)
237	VVDTPEIIHAQ	Lin et al. (2016)
238	EPAPPPEEKPRIK	Lin et al. (2016)
239	EKERIEAQ	Lin et al. (2016)
240	PPVDLEVHN	Lin et al. (2016)
241	APPPEEKPRIK	Lin et al. (2016)
242	VSPHGGPPEVPK	Lin et al. (2016)
243	SADEKTAIYK	Lin et al. (2016)
244	TLPIKDPHVDSA	Lin et al. (2016)
245	SSVFVVHPKES	Lin et al. (2016)
246	SSVFVVHPKE	Lin et al. (2016)
247	LPIKDPHVDS	Lin et al. (2016)
248	EAGPSIVHR	Lin et al. (2016)
249	TLPIKDPHVD	Lin et al. (2016)
250	SPKADFPH	Lin et al. (2016)
251	IAESQVNKL	Lin et al. (2016)
252	AEPPPPKEPEVPKK	Lin et al. (2016)
253	IESQPIVDTH	Lin et al. (2016)
254	SPRTPPPMQ	Lin et al. (2016)
255	GPDPIRYM	Lin et al. (2016)
256	IEEKSGMEGR	Lin et al. (2016)
257	ADEKTAIYK	Lin et al. (2016)
258	VGNEFVTK	Lin et al. (2016)
259	VIPEVTPPPKEEVVLK	Lin et al. (2016)
260	PGPVGPVGPAGAFGPRG	Lin et al. (2016)
261	GPVGPVGPAGAFGPRG	Lin et al. (2016)
262	LPIKDPH	Lin et al. (2016)
263	PGPVGPVGPAGAFGPR	Lin et al. (2016)
264	IGKGTPIPDLPEVK	Lin et al. (2016)
265	LIEDTEDWHPRTG	Lin et al. (2016)
266	FSAEEEFPDLSKHN	Lin et al. (2016)
267	KPIEVKGL	Lin et al. (2016)
268	KSKYTVVMDT	Lin et al. (2016)
269	LPIKDPHVD	Lin et al. (2016)
270	LTLDKVDVK	Lin et al. (2016)
271	PPDVEQAKK	Lin et al. (2016)
272	DQLDQLGMRMQH	Lin et al. (2016)
273	TEDWHPRTG	Lin et al. (2016)
274	KSEKERIEAQ	Lin et al. (2016)
275	LDKVDVK	Lin et al. (2016)
276	DEKTAIYK	Lin et al. (2016)
277	PAPAVQEDSRTF	Lin et al. (2016)
278	LFEATHGTAPKYAG	Lin et al. (2016)
279	TTPDTPEIRQ	Lin et al. (2016)
280	SSVFVVHPK	Lin et al. (2016)
281	SVLKDSALSTH	Lin et al. (2016)
282	VFVVHPKE	Lin et al. (2016)
283	TTPPKIDSPRA	Lin et al. (2016)
284	FAGDDAPRA	Lin et al. (2016)
285	RGDPGPVGPVGPAGAFGPRG	Lin et al. (2016)
286	GDPGPVGPVGPAGAFGPRG	Lin et al. (2016)
287	GDDNPVVH	Lin et al. (2016)
288	SPGIAGDPGPVGAP	Lin et al. (2016)
289	SSVFVVHPKESF	Lin et al. (2016)
290	AAAAPAPAPAPAPAPAPAKPKEPAIDLK	Lin et al. (2016)
291	TEPASRPPWVTDETFSQK	Lin et al. (2016)
292	TEGGETLTVKEDQVFS	Lin et al. (2016)
293	GGGYEVGFDAEYYRA	Lin et al. (2016)
294	DTEEVEHGEEEYEEEAHEAEEVHE	Lin et al. (2016)
295	TPVPASASYGESPAASTASKPRVVTT	Lin et al. (2016)
296	ILPRGAPVPPPASTSAYPTPVS	Lin et al. (2016)
297	GGGYEVGFDAEYYR	Lin et al. (2016)
298	WFNESKGFGF	Lin et al. (2016)
299	Niacinamide	Xiao, Ge, et al. (2019)
300	Pantothenate	Xiao, Ge, et al. (2019)
301	Riboflavin	Al‐Khalifa and Dawood (1993)
302	Thiamine	Yang et al. (2018)
303	Ethanol	Xiao, Ge, et al. (2019)
304	Isopropanol	Xiao, Ge, et al. (2019)
305	Myo‐Inositol	Xiao, Ge, et al. (2019)
306	3‐Hydroxybutyrate	Xiao, Ge, et al. (2019)
307	Triglyceride	Jayasena et al. (2013a)
308	Phospholipid	Jayasena et al. (2013a)
309	Acetoin	Pippen et al. (1960)
310	Diacetyl	Pippen et al. (1960)
311	Hexanal	Li et al. (2017)
312	Choline	Xiao, Ge, et al. (2019)
313	N‐Methylhydantoin	Xiao, Ge, et al. (2019)
314	O‐Acetylcarnitine	Xiao, Ge, et al. (2019)
315	Cholesterol	Choi (2011)
316	KOH	Nishimura et al. (2016)

^a^ C4:0, butyric acid; C6:0, caproic acid; C8:0, caprylic acid; C10:0, capric acid; C11:0, undecanoic acid; C12:0, lauric acid; C13:0, tridecanoic acid; C14:0, myristic acid; C15:0, pentadecanoic acid; C16:0, palmitic acid; C17:0, margaric acid; C18:0, stearic acid; C21:0, heneicosanoic acid; C22:0, behenic acid; C24:0, lignoceric acid; C14:1, myristoleic acid; C16:1, palmitoleic acid; C17:1, heptadecenoic acid; C18:1 n‐9t, vaccenic acid; C18:1 n‐9c, oleic acid; C18:2, linoleic acid; C18:3, α‐linolenic acid; C20:1, eicosenoic acid; C20:2, eicosadienoic acid; C20:3, eicosatrienoic acid; C20:4, arachidonic acid; C20:5, timnodonic acid; C22:1, erucic acid; C22:4, docosatetraenoic acid; C22:5, docosapentaenoic acid; C22:6, docosahexaenoic acid; C24:1, nervonic acid; 5′‐UMP, uridine 5′‐monophosphate; 5′‐CMP, cytidine 5′‐monophosphate; 5′‐GMP, guanosine 5′‐monophosphate; 5′‐AMP, adenosine 5′‐monophosphate; 5′‐IMP, inosine 5′‐monophosphate; 3′:5′‐cAMP, cyclic adenosine 3′:5′‐monophosphate; ATP, adenosine 5′‐triphosphate; ADP, adenosine 5′‐diphosphate; AMP, adenosine 5′‐monophosphate; GTP, guanosine 5′‐triphosphate; GDP, guanosine 5′‐diphosphate; GMP, guanosine 5′‐monophosphate; XMP, xanthosine 5′‐monophosphate; Pro‐Hyp, proline‐hydroxyproline; Hyp‐Gly, hydroxyproline‐glycine; and No. 178‐298: Peptide (single‐letter code) was identified in chicken/chicken soup.

**FIGURE 1:**
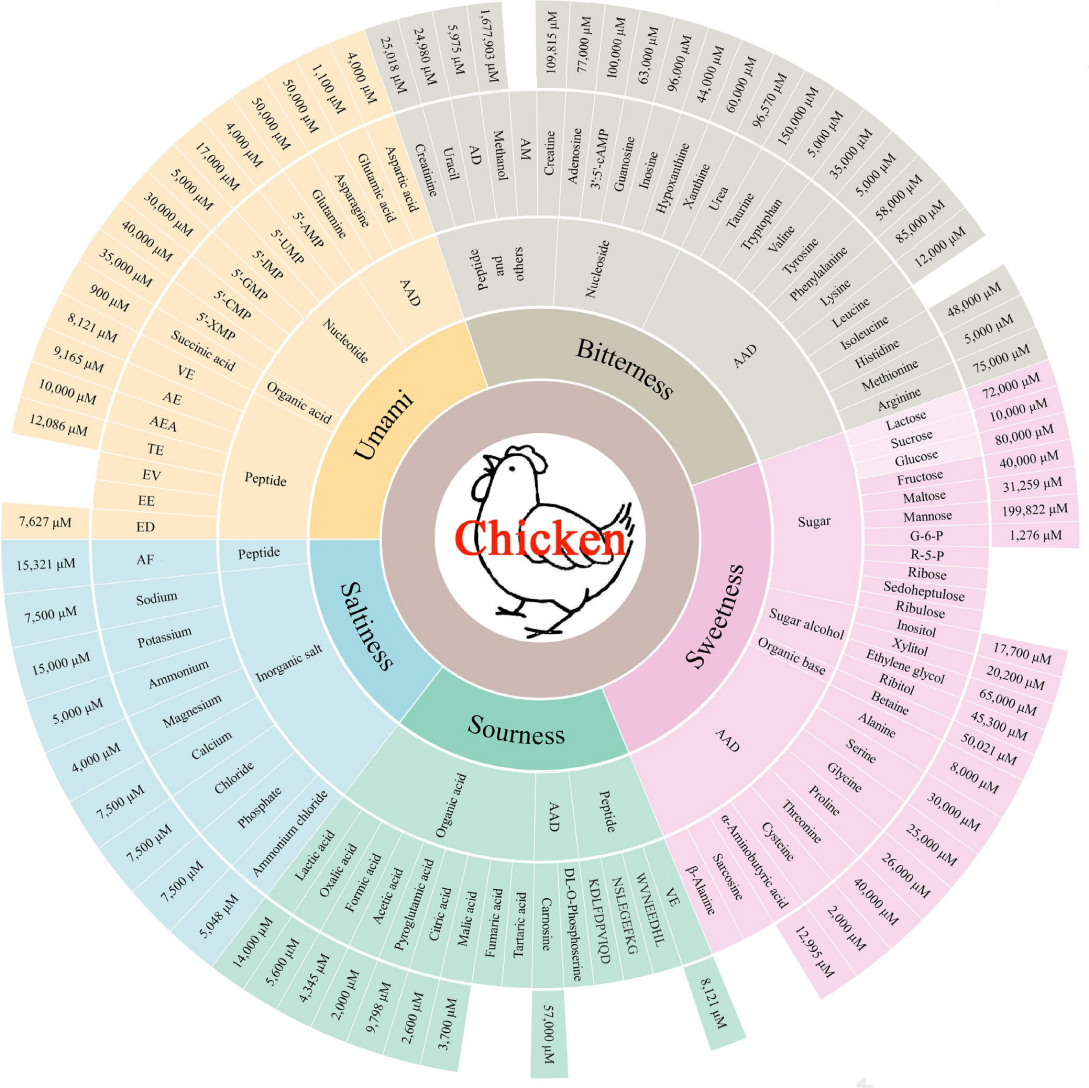
Taste‐active compounds and their taste threshold in chicken/chicken soup

The taste of chicken soup is attributed to the following: (a) chemical components transferred from the chicken carcass to the soup, including proteins, fats, carbohydrates, and minerals; and (b) the taste compounds produced by the reaction of water‐soluble taste precursors during heating. These reactions include the formation of flavor nucleotides, the thermal reaction of carbohydrates, the reaction of flavor peptides to amino acids, the reaction of reducing sugars to amino acids, and the oxidative degradation of lipids. In general, the main contributing components to the taste of chicken soup include sugars, amino acids, nucleotides, organic acids, salts, and peptides (Fukuuchi et al., 2018; Lin et al., 2016; Rikimaru & Takahashi, 2010). Figure 1 illustrates the taste compounds and their taste threshold values have been reported in chicken/chicken soup samples over the past 50 years.

### 2.1 Sweet‐tasting compounds

Sweet taste is one of the basic taste sensations and the most predominant preference among individuals. Natural sweet substances mainly include aliphatic hydroxyl compounds such as alcohols and sugars. However, amino acids, amides, lipids, and other compounds also have a sweet taste. The main sweet‐tasting compounds reported in chicken/chicken soup include sugars, sugar alcohols, and sweet amino acids. In the study reported by Dunkel and Hofmann (2009), 4 sugars (glucose, fructose, sucrose, and lactose), 4 sugar alcohols (inositol, xylitol, ethylene glycol, and ribitol), and 7 sweet‐tasting amino acids (alanine, serine, glycine, proline, threonine, cysteine, and methionine) were quantified in double‐boiled chicken broth. The taste threshold concentrations (TCs) of the mentioned above 15 sweet‐tasting compounds were also determined in bottled water by means of a triangle test. Among them, cysteine had the lowest taste threshold (TC = 2,000 μM), followed by methionine (TC = 5,000 μM) and alanine (TC = 8,000 μM). Since the side chain of methionine contains a hydrophobic methylthio group (CH_3_S‐), most researchers preferred to classify it as a bitter amino acid. In addition to inositol, glucose, and fructose, 4 free sugars (sedoheptulose, mannose (TC = 199,822 μM), ribose, and ribulose) were also identified in Single‐comb White Leghorn and New Hampshire chickens (Lilyblade & Peterson, 1962). The content of maltose (TC = 31,259 μM) was detected in the breast and thigh of Sanhuang chicken and Black‐bone silky fowl; however, the maltose content was found to be <0.1% in four samples (Wang et al., 2018). In the study reported by Aliani and Farmer, two phosphorylated (ribose‐5‐phosphate [R‐5‐P] and glucose‐6‐phosphate [G‐6‐P]) sugars were quantified, which showed that their content in breast meat was higher than that in leg meat (Aliani & Farmer, 2002, 2005). Recently, two sweet‐tasting amino acid derivatives (sarcosine and β‐alanine) were studied in chicken (Hy‐line brown) soup (Zhang et al., 2020); however, the contribution of these two compounds to the sweetness of chicken soup is unclear due to the lack of data on their taste threshold. Alkaloid is a type of basic organic compound that contains nitrogen. Almost all alkaloids have a bitter taste, except betaine that has a sweet taste. It was found that the content of betaine (CT = 50,021 μM) in freeze‐dried chicken soup prepared with Korean native chicken (KNC) was significantly higher than that prepared with commercial broiler (CB) (Jayasena et al., 2014).

### 2.2 Sour‐tasting compounds

Sour taste is one of the earliest chemical taste senses in animal evolution. Sourness provides a refreshing and exciting feeling and increases appetite. The formation of sourness is due to the dissociation of hydrogen ions (H^+^) from acidic compounds in an aqueous solution, which stimulates the taste receptors in the mouth. Then, it transmits signals to the taste center of the brain through the sensory nervous system. Organic acids are the main sour‐tasting compounds present in chicken/chicken soup. Four sour‐tasting compounds (lactate, malate, citrate, and acetate) were quantified in double‐boiled chicken broth (Dunkel & Hofmann, 2009). Among them, the taste threshold of acetate was the lowest (TC = 2,000 μM), followed by citrate (TC = 2,600 μM) and malate (TC = 3,700 μM). The contents of oxalic acid (TC = 5,600 μM), tartaric acid, formic acid (TC = 4,345 μM), pyroglutamic acid (TC = 9,798 μM), and fumaric acid were also determined in chicken/chicken soup (Horio & Kawamura, 1990; Norris et al., 1984; Stark et al., 2006; Zhang et al., 2020). Presently, the sour‐tasting peptides reported in chicken or chicken soup include VE (TC = 8,121 μM), WVNEEDHL, NSLEGEFKG, and KDLFDPVIQD (Kong et al., 2017; Zhang, Ma, et al., 2019).

### 2.3 Salty compounds

Saltiness is the characteristic taste of neutral salt, which plays an essential role in cooking. As cations are easily adsorbed by the carboxyl group or phosphoric acid group of the taste receptor proteins, the salty taste perceived by the tongue is mainly attributed to metal cations, such as sodium ions and potassium ions. The metal cations in chicken/chicken soup include Na^+^, K^+^, ${\text{NH}}_{4}^{+}$, Ca^2+^, and Mg^2+^ (Choi, 2011). As the taste of divalent salts is complex and mainly characterized by bitterness and saltiness, the taste TC of CaCl_2_ and MgCl_2_ was found to be bitter in the study reported by Dunkel & Hofmann (2009; Lawless et al., 2003). The contribution of anions to saltiness has long been proven (Elliott & Simon, 1990; Roebber et al., 2019). The study conducted by Lawless et al. (2003) have revealed that salts with larger anions are not effective stimulants because of their limited expansion at the tight junction and at the basolateral area of taste receptor channels. The anions reported in chicken/chicken soup mainly included Cl^−^, OH^−^, ${\text{HPO}}_{4}^{2‐}$, and ${\text{PO}}_{4}^{3‐}$ (Nishimura et al., 1988, 2016; Qi et al., 2017).

### 2.4 Umami‐tasting compounds

As the characteristic taste of chicken soup, umami is an important evaluation index of high‐quality chicken soup. The umami‐tasting compounds in chicken/chicken soup mainly include umami‐tasting amino acids, 5′‐nucleotides, and umami peptides. In the study reported by Dunkel and Hofmann (2009), 4 amino acids (glutamic acid, aspartic acid, glutamine, and asparagine), 1 organic acid (succinic acid), and 6 nucleosides (adenosine 5′‐monophosphate [5′‐AMP], uridine 5′‐monophosphate [5′‐UMP], guanosine 5′‐monophosphate [5′‐GMP], xanthosine 5′‐monophosphate [5′‐XMP], cytidine 5′‐monophosphate [5′‐CMP], and inosine 5′‐monophosphate [5′‐IMP]) were quantified and their taste threshold was determined in double‐boiled chicken broth. Among them, the taste threshold of succinic acid was the lowest (TC = 900 μM), followed by glutamic acid (TC = 1,100 μM) and aspartic acid (TC = 4,000 μM). Several studies have shown that 5′‐IMP is responsible for the umami taste of chicken meat. The taste threshold of 5′‐IMP in bottled water was 5,000 μM (Dunkel & Hofmann, 2009). The contents of 5′‐IMP in chicken soup vary with the individual sample (variety, age, gender, and so on), feed nutrition, tissue position, cooking method, storage method, and evaluation method. The umami peptide is another type of umami‐tasting compound considered after free amino acids, nucleotides, and organic acids. As early as 1999, Maehashi et al. isolated the umami peptides, EE and EV, from the chicken protein hydrolysate. In addition to these two umami peptides, four other peptides (ADE, AED, DEE, and SPE) were proven to enhance the umami intensity of 5′‐IMP (Maehashi et al., 1999). Presently, the umami peptides reported in chicken/chicken soup included AH, VE (8,121 μM), AE (9,165 μM), ED (7,627 μM), AEA (10,000 μM), TE (12,086 μM), WVNEEDHL (umami‐enhancing), NSLEGEFKG (umami‐enhancing), and KDLFDPVIQD (umami‐enhancing) (Kong et al., 2017; Zhang, Zhao, et al., 2019).

### 2.5 Bitter‐tasting compounds

The bitter taste is easily perceived and lasts for a longer duration in the mouth. The taste threshold of caffeine was only 750 μM (Stark et al., 2006). Bitter taste exerts a double effect on the quality of chicken soup. An ideal extent of bitter taste in chicken soup can help enrich and improve the taste of chicken soup. However, a considerable extent of bitter taste will affect the taste of chicken soup and result in consumer dissatisfaction. The bitter‐tasting compounds in chicken/chicken soup mainly include bitter amino acids, nucleotides, and bitter peptides. Strong hydrophobic sites reportedly exist in the molecular structure of several bitter organic compounds. The intensity of the hydrophobic interaction between hydrophobic sites and taste cell membrane may help determine the extent of bitterness (Katsuragi et al., 1996). In Dunkel and Hofmann′s study, 16 bitter‐tasting amino acids and nucleosides (arginine, histidine, isoleucine, leucine, lysine, phenylalanine, tyrosine, valine, tryptophan, taurine, xanthine, hypoxanthine, inosine, guanosine, adenosine, and 3′:5′‐cAMP) were quantified in double‐boiled chicken broth. Among them, tyrosine and tryptophan had the lowest taste thresholds (TC = 5,000 μM), followed by isoleucine (TC = 11,000 μM) and leucine (TC = 12,000 μM) (Dunkel & Hofmann, 2009). The bitter‐tasting methionine and creatine contents in freeze‐dried chicken soup prepared using CB were significantly higher than those prepared using KNC (Jayasena et al., 2015). The bitter peptides, AM and AD (5,975 μM), were separated and identified in chicken enzymatic hydrolysate (Kong et al., 2017). Owing to unclear quantitative information on the two peptides, their contribution to the bitterness of chicken enzymatic hydrolysate could not be ascertained. Additionally, the bitter‐tasting compounds, namely urea (96,570 μM), methanol (1,677,903 μM), uracil (24,980 μM), and creatinine (25,018 μM), were also observed in chicken/chicken soup (Xiao, Ge, et al., 2019; Zhang et al., 2020).

## 3 EVALUATION METHODS

### 3.1 Sensory evaluation

Sensory evaluation using the human tongue as a detector can provide direct, effective, unique, and intuitive taste information for chicken soup. Moreover, it provides a human feeling and is considered to be the most direct and intuitive tool for such evaluation. The detection threshold of the human tongue (45 years of age and below) for sodium chloride, quinine sulfate, sucrose, and citric acid was reported to be 2.49 × 10^–3^ M, 1.24 × 10^–6^ M, 5.92 × 10^–3^ M, and 1.04 × 10^–4^ M, respectively (Weiffenbach et al., 1982). Thereby sensory evaluation becomes a valuable and sensitive method in analyzing chicken soup taste and taste‐active compounds. The sensory evaluation of chicken soup is usually conducted by directly tasting the warm chicken soup (45–60°C). A sensory panel often includes 6–19 well‐trained panelists. Linear scale (Li et al., 2017), category scale (Kurobayashi et al., 2008), and quantity scale (Kong et al., 2017) are widely used to evaluate the taste attribute strength of chicken soup.

Sensory evaluation is mainly performed to distinguish between the taste characteristics of different chicken soup or to score the taste attributes of chicken soup. The five sensory attributes (chicken meat‐like, fatty, off‐flavor, kokumi, and umami) of chicken soup prepared using traditional clay stew‐pot and commercial ceramic electrical stew‐pot could be distinguished based on the taste sensed (Zhang et al., 2018). The overall acceptance of chicken soup subjected to refrigerated (4 ± 1°C) and superchilled (−2.5 ± 1°C) storage conditions could be identified by the human tongue (Li et al., 2017). Umami and saltiness of chicken meat after stewing for 1 and 2 hr could be distinguished based on the taste sensed (Qi et al., 2018). The umami, saltiness, and sweetness of chicken broth containing a nonvolatile fraction of celery (7%) and chicken broth (control) could also be identified by the human tongue (Kurobayashi et al., 2008). The sensory evaluation of chicken soup taste analysis is fast; however, it is inevitably affected by the physiological, physical, psychophysical, and other factors of panelists. The sensory abilities of human tongue markedly vary among different individuals. Additionally, sensory evaluation is nonquantitative and markedly affected by individual subjectivity. Sensory fatigue, a source of error, also restricts the efficiency of intensive evaluation. Therefore, effective technical training of panelists, uniformity of the description, and consideration of reference standards for chicken soup taste are of significance in sensory evaluation.

The quantitative descriptive analysis (QDA) method was performed to analyze the taste characteristics of chicken soup. In the QDA method, panelists were requested to describe the perceived sensory attributes and the intensities of the attributes. Many descriptive words have been reported, including taste, flavor, and mouthfeel (You et al., 2019; Zhang et al., 2018) (Table 2), rendering the descriptive analysis more accurate and consistent. The descriptive words covered both positive taste attributes (sweet, salty, umami, chicken meat‐like, fatty, meaty, and kokumi) and negative ones (bitter, sour, greasy, rubbery, and warm‐over flavor) (Hooge & Chambers, 2010; Miyaki et al., 2015; Zhan et al., 2020; Zhang et al., 2018). The reference standard used for the identification of taste attributes is not unique. For example, caffeine and quinine were used to describe bitterness. The type of reference scale used in the QDA method influences the result. The partial least squares regression correlation model using three sensory descriptors (chicken meat‐like, fatty, and off‐flavor) was established (Zhang et al., 2018). This model established an association between flavor‐active compounds and sensory data, which aided the prescreening of flavor‐related compounds in chicken soup (Zhang et al., 2018). The results of Pearson correlation analysis and principal component analysis (PCA) both show that the taste attributes of chicken soup are significantly correlated with 14 taste compounds (inosine, GMP, sarcosine, α‐aminobutyric acid, valine, leucine, asparagine, methionine, α‐aminoadipic acid, ornithine, lysine, histidine, tryptophan, and cystine) (Pérez‐Palacios et al., 2017). Although the QDA method has been widely used in chicken soup taste evaluation, the unification of taste standards and scientific training remain the focus of future research.

**TABLE 2:** Descriptive sensory analysis attributes and references used to evaluate chicken soup

Sensory attributes	Definitions	References intensity	Source
Taste			
Chicken meat‐like	–	100 g lean minced chicken meat was cooked in water at 100°C for 2 hr	Zhang et al. (2018)
Fatty	–	100 g refined chicken oil was heated at 100°C for 2 hr	Zhang et al. (2018)
	–	refined tallow, extracted from chicken fat	Zhan et al. (2020)
	–	0–10 mM chicken fat (Score: 0–10)	You et al. (2019)
Meaty	–	Chicken brisket (0.5 kg, 2.5 cm thick) boiled in water for 2 hr	Zhan et al. (2020)
Greasy	–	100 g of chicken oil boiled in water at 100°C for 4 hr	Zhang et al. (2018)
Rubber and warm‐over flavor	–	100 g of chicken oil in boiling water for 10 min and then stored at 4°C for 3 day	Zhang et al. (2018)
Umami	–	The taste associated with 0.15% monosodium glutamate (MSG)	Zhang et al. (2018)
	–	1.5 g/L MSG	Zhan et al. (2020)
	One of the basic taste, common to MSG. The taste and mouth‐filling sensation of compounds such as glutamates that is savory, brothy, meaty, rich, full, and complex, common to many foods such as soy sauce, stocks, ripened cheese (especially parmesan), shellfish (crab, lobster, scallops, clams), mushrooms (especially porcini), ripe tomatoes, cashews, and asparagus	Kitchen Basics chicken broth (Score: 2) 0.5% MSG in Kitchen Basics chicken broth (Score: 3.5)	Miyaki et al. (2015)
	–	0.08 g MSG/100 ml in water (Score: 5) 0.16 g MSG/100 ml in water (Score: 10)	Zhang et al. (2020)
	Perceived as umami taste.	–	Kurobayashi et al. (2008)
	–	0–10 mM MSG (Score: 0–10)	You et al. (2019)
	–	0–20 mM blank chicken soup (Score: 0–10)	You et al. (2019)
Saltiness	A basic taste of which the taste of sodium chloride in water is typical	2.25 g NaCl in 500 ml filtered water (Score: 7.5) 2.75 g NaCl in 500 ml filtered water (Score: 10.0) 3.10 g NaCl in 500 ml filtered water (Score: 12.5)	Hooge and Chamber (2010)
	One of the basic taste, common to sodium chloride	0.2% NaCl in water (Score: 2) 0.5% NaCl in water (Score: 5)	Miyaki et al. (2015)
	–	0.3 g NaCl/100 ml in water (Score: 5) 0.6 g NaCl/100 ml in water (Score: 10)	Zhang et al. (2020)
	Perceived as salty taste	–	Kurobayashi et al. (2008)
Sweetness	A basic taste of which the taste of sucrose in water is typical	10.0 g sucrose in 500 ml filtered water (Score: 2.0) 25.0 g sucrose in 500 ml filtered water (Score: 5.0)	Hooge and Chamber (2010)
	–	2 g sucrose/100 ml water (Score: 5) 4 g sucrose/100 ml water (Score: 10)	Zhang et al. (2020)
	Perceived as sweet taste	–	Kurobayashi et al. (2008)
	–	0–50 mM sucrose (Score: 0–10)	You et al. (2019)
Sourness	A basic taste of which the taste of citric acid in water is typical	0.25 g citric acid in 500 ml filtered water (Score: 2.0) 0.40 g citric acid in 500 ml filtered water (Score: 5.0)	Hooge and Chamber (2010)
	–	0.05 g citric acid/100 ml water (Score: 5) 0.1 g citric acid/100 ml water (Score: 10)	Zhang et al. (2020)
Bitterness	A basic taste of which the taste of caffeine in water is typical	0.25 g caffeine in 500 ml filtered water (Score: 2.0) 0.40 g caffeine in 500 ml filtered water (Score: 5.0)	Hooge and Chamber (2010)
	–	0.00075 g quinine/100 ml water (Score: 5) 0.0015 g quinine/100 ml water (Score: 10)	Zhang et al. (2020)
	–	0–1 mM caffeine (Score: 0–10)	You et al. (2019)
Kokumi	–	chicken broth with added 3 mM glutathione	Zhang et al. (2018)
	–	3 mM glutathione	Zhan et al. (2020)
Total aftertaste	The total aftertaste intensity after 5 s of all flavor notes within the sample	No reference	Miyaki et al. (2015)
Flavor			
Total flavor	The total intensity of all of the flavors of the sample including basic tastes	Kitchen Basics chicken broth (Score: 6)	Miyaki et al. (2015)
Total chicken/meaty flavor	The flavor intensity reminiscent of cooked chicken meat	Kitchen Basics chicken broth (Score: 5)	Miyaki et al. (2015)
Chicken flavor	The flavor intensity reminiscent of cooked chicken	Kitchen Basics chicken broth (Score: 5)	Miyaki et al. (2015)
Bones/marrow flavor	The character associated with chicken bones, particularly the marrow of chicken bones	No reference	Miyaki et al. (2015)
Roasted flavor	The total flavor intensity that is reminiscent of roasted chicken and/or vegetables	Swanson′s chicken broth (Score: 6)	Miyaki et al. (2015)
Total vegetable flavor	The total flavor intensity of vegetables such as carrots, green vegetables, and herbs in the broth	Kitchen Basics chicken broth (Score: 5)	Miyaki et al. (2015)
Richness	The degree to which the flavor characters of the sample are harmonized, balanced, and blend well together as opposed to being spiky or striking out	No reference	Miyaki et al. (2015)
Thick	Generous, deep, rich	–	Kurobayashi et al. (2008)
Impactful	Impressive, characteristic	–	Kurobayashi et al. (2008)
Mild	Mellow, harmonious, balancing	–	Kurobayashi et al. (2008)
Lasting	Mouthfulness, continuity, aftertaste	–	Kurobayashi et al. (2008)
Satisfied	Impressive, mellow, full	–	Kurobayashi et al. (2008)
Complex	Mixed	–	Kurobayashi et al. (2008)
Refined	Sophisticated, elegant	–	Kurobayashi et al. (2008)
Clarified	Reduced unfavorable meaty or fatty	–	Kurobayashi et al. (2008)
Mouthfeel			
Viscosity	The degree to which the samples are viscous in the mouth from thin to thick	Water (Score: 1) Heavy whipping cream (Score: 6)	Miyaki et al. (2015)
Mouthfulness	The perception that the sample fills the whole mouth is blooming, or growing, a full‐bodied sensation when the sample is held in the mouth	Kitchen Basics chicken broth (Score: 1.5) 0.5% MSG in Kitchen Basics chicken broth (Score: 3)	Miyaki et al, (2015)
Mouth coating	The degree to which there is a leftover residue, a slick, powdery, or fatty coating or film in the mouth that is difficult to clear.	0.5% MSG in water (Score: 4) Half and half (Score: 5)	Miyaki et al. (2015)
Tongue coating	The degree to which there is a leftover residue, a slick, powdery, or fatty coating or film on the tongue that is difficult to clear	0.5% MSG in water (Score: 3)	Miyaki et al. (2015)
Total trigeminal	The intensity of the total sensation, including numbing, burning, tingling, or irritation, impaired on the soft tissues of the oral cavity, particularly the tongue	Wintergreen breathsaver (not scored) 0.5% MSG in water (Score: 5)	Miyaki et al. (2015)
Salivating	The degree to which the sample caused a perceived increase in salivation	No reference	Miyaki et al. (2015)
Swelling of cheeks and lips	The feeling of swelling of the soft tissue in the oral cavity, specifically the cheeks and lips, reminiscent of the perception of swelling produced by antithetic treatments at a dental office, but without a distinct numbing effect	0.5% MSG in water (Score: 4)	Miyaki et al. (2015)

### 3.2 Instrumental detection

#### 3.2.1 Electronic tongue

Electronic tongue, a tool used to evaluate food taste by simulating human taste, is based on sensor array, signal acquisition, and pattern recognition systems (Makkliang et al., 2015). The electronic tongue uses a material similar to that observed in biological systems as a sensitive membrane of the sensor. When one side of the lipid film establishes contact with the taste substance, the membrane potential changes, resulting in a response and the detection of the relationship between various substances (Makkliang et al., 2015; Xiao, Tahara, et al., 2019). Electronic tongue, a simple and rapid tool, provides global taste perception for soluble taste compounds in chicken soup samples. It is mainly used to analyze the difference among chicken soup samples because it can rapidly distinguish between and quantify the different taste senses in the sample. Furthermore, it can be used to detect subtle changes in chicken soup samples. Electronic tongue combined with PCA data processing was used to investigate the effects of stewing time (1, 2, and 3 hr) on traditional Chinese chicken soup (Qi et al., 2017). The electronic tongue could successfully distinguish between three types of chicken soup samples with high accuracy (Qi et al., 2017). The extract of Dezhou braised chicken in different processing stages was analyzed, and the radar fingerprint was formed according to the output data of 8 taste sensors. The electronic tongue could thus be applied to explore the evolution of different taste components in Dezhou stewed chicken (Liu et al., 2017). The electronic tongue could also be used to estimate the degree of adulteration (Tian et al., 2019). When the chicken was mixed with mutton in proportions of 0%, 20%, 40%, 60%, 80%, and 100%, the meat extract was detected using the electronic tongue (Tian et al., 2019). Results showed that the electronic tongue with least squares support vector machines (LS‐SVM) was the most effective method for predicting chicken content (Tian et al., 2019). Of note, all data obtained by using the electronic tongue are analyzed as a whole, and detailed taste‐active compound information is not required. Therefore, the use of the electronic tongue has advantages in identification and classification but exhibits limitations in quantitative analysis. Furthermore, the selectivity and limitations of the sensors prevent their widespread application in food evaluation.

#### 3.2.2 Liquid chromatography method

The liquid chromatography (LC) technology is the most common and effective method for conducting chicken soup taste analysis. In fact, it is widely used for performing qualitative and quantitative analysis of taste‐active compounds in chicken soup. The complex composition of chicken soup, with insufficient concentration of certain taste compounds, requires higher sample pretreatment requirements. High‐speed centrifugation, filtration, ultrafiltration, selective precipitation, extraction, derivatization, concentration (rotoevaporation, freeze‐drying, and so on), and other technologies are widely used in the preprocessing stage of samples (Kong et al., 2017; Qi et al., 2018; Wang et al., 2018; Zhang et al., 2020). In the determination of water‐soluble taste substances in chicken soup, degreasing is an inevitable operation step used for sample pretreatment. Additionally, the removal of macromolecular proteins is necessary to protect the chromatographic column. Perchloric acid and methanol are commonly used for protein precipitation in chicken soup samples (Zhang et al., 2020). For the identification of unknown taste compounds (such as peptides), most are subjected to ultrafiltration separation, gel filtration separation, and high‐performance liquid chromatography separation to obtain relatively pure compounds for conducting further qualitative and quantitative analysis (Kong et al., 2017; Zhang, Zhao, et al., 2019).

The mass spectrometer (MS) is a commonly used mass detector. The combination of separation technology and MS helps to obtain a wealth of compound information (including molecular weight and structure information) in a sample. Additionally, the characteristics of high specificity and sensitivity have important contributions to the qualitative and quantitative analysis of compounds. Instead of conducting exploration and identification of several compounds, mass spectrum data could be analyzed as a sample “fingerprint” from a whole sample. The lipid profiles of Taihe and crossbred black‐boned silky fowls were analyzed using ultra‐performance liquid chromatography–tandem mass spectrometry (UPLC‐MS/MS) at a mass scan mode, and the sample “fingerprint” formed by quantified lipids was used for conducting discrimination by statistical analysis (Mi et al., 2018). The orthogonal partial least squares discriminant analysis results showed that Taihe and crossbred black‐boned silky fowls could be effectively distinguished from each other (Mi et al., 2018). Based on a study conducted on the effects of quercetin and cinnamaldehyde on the chemical composition of beef soup, 636 molecular features were identified by performing UHPLC‐MS/MS (Li et al., 2020). Compared to databases based on data obtained by gas chromatography, most databases based on liquid chromatography data are nonuniversal. Furthermore, a database for taste compounds has not been established. Thus, the engagement of remarkable efforts is necessary for data processing.

#### 3.2.3 Nuclear magnetic resonance

Nuclear magnetic resonance (NMR) is a nondestructive and rapid method used for the identification and characterization of small molecules. For the past few years, NMR techniques have been rapidly developed and it has been successfully used for the analysis of chicken quality. The focus of NMR studies includes the estimation of the meat quality variation caused by raw materials (variety (Liu et al., 2019; Wang et al., 2016), age (Xiao, Ge, et al., 2019), and position (Xiao, Ge, et al., 2019)) and external conditions (storage (Graham et al., 2010; Li et al., 2014), and processing (Li et al., 2000; Shaarani et al., 2006)). Visual analysis of the correlation between taste precursors and chicken samples was achieved using NMR and multivariate data analysis techniques (PCA and partial least squares discriminant analysis [PLS‐DA]). Xiao, Ge, et al. (2019) studied the taste precursors in Wuding chickens at five ages and compared the taste components of chicken breast and thigh meat. The results showed that there were eight taste compounds in 230‐day‐old chickens, which were significantly different from the other four age groups. Furthermore, organic acids and small peptides were identified as the main taste precursors of chicken breast and thigh meat (Xiao, Ge, et al., 2019). The ^1^H‐NMR results showed that boiling treatment had a significant effect on the distribution of water‐soluble low‐molecular‐weight compounds in Wuding chicken (Xiao, Luo, et al., 2019). NMR, mainly used to analyze polar metabolites, permits the detection of a broad scope of analytes within a single run. However, NMR typically exhibits disadvantages such as lower sensitivity compared to GC‐MS and LC‐MS (Lei et al., 2011; Ruiz‐Aracama et al., 2012).

#### 3.2.4 Gas chromatography–mass spectrometry

Gas chromatography–mass spectrometry (GC‐MS) is typically used to analyze (semi‐)volatile compounds and active aroma ingredients in chicken soup (Feng et al., 2018), due to its robustness, reproducibility, and selectivity, and owing to the availability of a considerable number of commercial and "in‐house" databases. In certain studies, the derivatization of polar compounds has expanded the scope of this approach, thus highlighting it as one of the most effective, repeatable, and widely used analytical platforms in taste‐active compound research (Beale et al., 2018). In the food industry, the GC‐MS combined with derivatization has been used for the quantitative analysis of taste‐active compounds (amino acids and their derivatives, organic acids, sugars and sugar alcohols, etc.) in the fermentation of soybean paste (Sun et al., 2019), rice *koji* (Lee et al., 2016), and so forth. However, this method is rarely performed to assess the taste‐active compounds in chicken soup. This may be due to the complexity of derivatization. During the addition of appropriate derivatization reagents, it is important to ensure the transformation of all target compounds to their derivatives to confirm that the corresponding peaks do not dominate the total ion chromatography peaks or mask other peaks (Beale et al., 2018).

#### 3.2.5 Others

In addition to the above‐mentioned techniques, the ambient desorption ionization techniques have been used to assess chicken quality. This technique is a modern approach for metabonomics fingerprint and profile analysis, which can be used for direct sample detection in an open environment with high sample throughput. As a new atmospheric pressure ion source, real‐time ionization mass spectrometry (DART‐MS) is independent of size and morphology and does not require the conduction of pretreatment steps. Cajka et al. proposed a rapid method for metabolomic fingerprinting of chicken muscle and feed using real‐time (DART) ion source coupled to a medium‐high resolution/accurate mass time‐of‐flight mass spectrometer (TOF‐MS), followed by multivariate data analysis of the acquired data sets, which highlighted the traceability of chicken quality and feed materials (Cajka et al., 2013).

## 4 FACTORS AFFECTING CHICKEN SOUP TASTE

### 4.1 Genetic factors

The taste of chicken is a markedly inherited trait. The genes controlling the taste trait have been studied. Based on findings reported by previous studies, the taste trait deemed is relatively complex and genetically controlled. There are different genes involved in the synthesis of taste compounds in chicken. 5′‐IMP, as an essential index for meat flavor determination (Fujimura, 1998; Kawai et al., 2002), has been systematically studied in the literature. The *GPAT, AIRC, PurH, GARS‐AIRS‐GART, ADSL*, and *AMPD1* genes were identified as the most common candidate genes for 5′‐IMP content in chicken.

*GPAT* and *AIRC* encode two enzymes that catalyze step 1 and steps 6 plus 7, respectively, of the de novo purine biosynthetic pathway (Zhang et al., 2009). The chicken *GPAT/AIRC* genes are located on chromosome 6. The *PurH* gene is responsible for encoding the attic enzyme, a 64‐kDa bifunctional enzyme that catalyzes the final two reactions in de novo purine biosynthesis and possesses two enzymes, namely AICAR transformylase and 5′‐IMP cyclohydrolase (Asby et al., 2015). The crystal structure of chicken ATIC showed the absence of an intermediate connecting channel between the N‐extremity and C‐extremity activity centers, which plays an important regulatory role in the synthesis of 5′‐IMP (Greasley et al., 2001). Shu et al. (2010) showed that the *GPAT/AIRC* and *purH* genes affected muscle 5′‐IMP content. As a result, they might be candidate loci or linked to major genes that affect muscle 5′‐IMP content; the epistatic effects were found to be higher than the single genotype effects in Chinese Baier chicken. Three SNPs in *GPAT* gene (exon 2), *AIRC* gene (exon 3 and 8), *pur*H gene (exon 16), and *GPAT/AIRC* (promoter region) were associated with muscle 5′‐IMP content in chickens (Shu et al., 2008, 2010).

The *GARS‐AIRS‐GART* genes are located on chromosome 1, which catalyze steps 2, 3, and 5, respectively, of the de novo purine biosynthetic pathway. Chicken *GARS‐AIRS‐GART* gene has been studied as a candidate gene for determination of the effects on the 5′‐IMP content in the muscle (Shu et al., 2007; Ye et al., 2010). Furthermore, a favorable association between genotypes and higher 5′‐IMP content has been demonstrated in several Chinese native chicken breeds.

The *ADSL* gene plays an important role in the biological pathways of purine nucleotide de novo synthesis, and its mutation can reduce the ability of 5′‐IMP synthesis in vivo (Lundy et al., 2010). Studies have revealed no significant correlation between the content of exogenous 5′‐IMP and the expression of *ADSL*. This may be attributable to the purine biosynthetic pathway that is ultimately responsible for the generation of 5′‐IMP from α‐D‐ribose‐5‐phosphate (Zhang et al., 2008).

The *AMPD1* gene is primarily expressed in muscle tissues and is involved in the metabolism of 5′‐IMP. Hu et al. revealed that animals with the homozygous genotype AA at positions 4,064 and 6,805 presented with significantly higher 5′‐IMP contents than those with the GG genotype (*p* < .05). Further, they found that the homozygous genotype AA at position 6,805 resulted in a significantly higher 5′‐IMP content than the genotype GG for both cock and hen (Hu et al., 2015). Chen et al. (2008) estimated the genetic parameters of 1,069 purebred Beijing‐You full‐sib male chickens and found that the heritability of muscle 5′‐IMP content was moderate (0.23) (Chen et al., 2008). Therefore, 5′‐IMP content in chicken meat could be increased through genetic selection. Owing to the adequately high heritability of breast and fat yield characteristics (Le Bihan‐Duval et al., 1998), poultry body composition has been substantially improved by selection.

### 4.2 Preslaughter factors

Many studies have shown that under the same cooking conditions, factors such as chicken breed, age, sex, and diet affect the content of taste substances in chicken and thus affect the quality of chicken soup (Jayasena et al., 2013b; De Zwart et al., 2003). In Korea, KNC is more popular among consumers because of its characteristic flavor and texture compared to CB (Jayasena et al., 2013b). As the production of KNC is insufficient to meet consumer demand (Jeon et al., 2010), traditional dishes such as samgyetang and baeksuk are made with CB instead of KNC. Studies are underway to clarify the effect of chicken breed on the quality of soup, which is important for maintaining the characteristics of chicken soup. The analysis of the taste components in defatted freeze‐dried chicken soup (DFDS) prepared with KNC and CB showed that the DFDS prepared with KNC had higher contents of 5′‐IMP, betaine, inosine, cysteine, and carnitine (Jayasena et al., 2015). Additionally, the lipid layer isolated from KNC soup showed significantly higher levels of linoleic (C18:2), α‐linolenic (C18:3), arachidonic (C20:4), and docosahexaenoic acid (C22:6, DHA) and low saturated fatty acid (Choe et al., 2010; Jeon et al., 2010). In general, the DFDS prepared using KNC was superior to the DFDS prepared using CB in terms of nutrition and sensory quality (Jung et al., 2011).

The sex of KNC also significantly influences the taste characteristics of freeze‐dried broth (FDB) (Jayasena et al., 2014). A study conducted on the taste compounds of FDB samples prepared using 100‐day‐old male and female KNC showed that the 5′‐IMP and arachidonic acid (C20:4) contents in samples prepared using female FDB were significantly higher than those prepared using male FDB. The FDB samples prepared using male KNC contained higher levels of inosine, linoleic (C18:2), glycine, alanine, lysine, and serine (*p* < .05). However, the levels of betaine, carnitine, glutamic acid, creatine, oleic acid, and docosahexaenoic acids (C22:6; DHA) in FDB were not significantly different between the two sexes (*p* > .05) of KNC.

The content of taste‐active compounds in DFDS was significantly affected by the age of KNC (Jayasena et al., 2015). The study of taste‐active compounds in DFDS prepared with KNC of 5 ages (10, 11, 12, 13, and 14 weeks of age) revealed that inosine and linoleic acid contents in DFDS increased with an increase in age. In contrast, the contents of 5′‐AMP, oleic acid, and hypoxanthine decreased. The 5′‐IMP content of DFDS fluctuated significantly with the increase in KNC age. Studies conducted by Jayasena et al. revealed that age exerted a significant effect on the content of oleic acid, arachidonic acid, and DHA in KNC meat. The content of free amino acids responsible for the umami taste (glutamic acid and aspartic acid), sweet taste (alanine, serine, and glycine), and bitter taste (valine, isoleucine, leucine, phenylalanine, methionine, arginine, and histidine) decreased significantly as the age of KNC increased. However, the age of KNC exerted a positive effect (*p* < .05) on lysine content that is also responsible for the sweet taste. Therefore, the effect of age on the content of 5′‐IMP and glutamate in meat depends on the state of slaughter and cooking status (Jayasena et al., 2015). In cooked KNC leg meat, a positive correlation between bird age and reducing sugar content (*p* < .05) was observed. However, in KNC breast, there was a negative correlation between age and DHA content, especially after 13 weeks.

Studies have shown that dietary nutrients play a significant part in determining taste compounds of chicken meat (Fujimura and Kadowaki, 2006). Fujimura and Kadowaki (2006) reported that free glutamic acid and sensory score in chicken meat were increased in high crude protein diet. The free glutamic acid content in chicken muscle was negatively correlated with the level of leucine in the diet (Fujimura and Kadowaki, 2006). Laksesvela (1960) reported that the taste of chicken meat was significantly improved when the chicken were provided with 36.7 mg/kg of *d‐α*‐tocopheryl acetate. In addition, reducing dietary lysine content was proved to increase the contents of free glutamic acid, glycine, valine, isoleucine, leucine, histidine, and threonine content in chicken meat and significantly improved the umami and kokumi tastes of chicken meat (Watanabe et al., 2016).

Other preslaughter factors such as heat stress and preslaughter shackling also contribute to the taste quality of chicken. Ali et al. (2008) reported that chickens exposed to heat stress before slaughter showed the lowest limit pH, which had a negative impact on meat quality. The lactate concentration in chicken breast muscle was found to increase with struggling activity (Papinaho et al., 1995).

### 4.3 Processing

#### 4.3.1 Preprocessing

After subjection to slaughter, the circulatory system and oxygen ssupply of chicken tissues are terminated. As a result, metabolism conditions in the muscle tissues change from aerobic metabolism to anaerobic fermentation, during which lactic acid is produced and accumulated. The lactic acid content in chicken was found to reach the maximum value (6.75 mg/g muscle) within 1 day after slaughter and reduce gradually to 5.47 mg/g muscle at 3 days after slaughter (Nishimura et al., 1988). The lactic acid content in the heated chicken soup was higher under the condition of additional storage than that without additional storage. Considering the sourness and water solubility of lactic acid, it might contribute to the taste quality of chicken soup. Gault (1985) reported that lower pH value has a negative influence on the water‐holding capacity and tenderness of meat. In addition, the increase of acid condition in chicken muscle leads to denaturation of muscle protein. With the occurrence of meat stiffness, denatured proteins (such as sarcoplasma, myofibril, and myoglobin) are easily affected by proteases, and the N‐terminal of protein is separated one by one, forming a variety of low molecular peptide and amino acid compounds. Studies have shown that the levels of oligopeptides and all free amino acid increased with the degradation of protein during storage, which were positively correlated with the taste of chicken soup (Nishimura et al., 1988). Meanwhile, the ATP in the chicken muscle after slaughter could be degraded by the pathway ATP→ADP→AMP→IMP→HxR→Hx by the corresponding intracellular enzymes (Nakatani et al., 1986), and its metabolite 5′‐IMP is a kind of compound which has important contribution to the umami taste of chicken. Studies have shown that the 5′‐IMP content of chicken breast muscle reached a maximum at 8 hr after slaughter and then decreased gradually (Nishimura et al., 1988). Although the level of 5′‐IMP in the heated soup of meat after additional storage was less, the importance of 5′‐IMP in meat taste should be studied in detail, considering its synergistic effect with glutamic acid.

Immersion in hot water is one of the strategies for reducing the abundance of pathogenic bacteria on the surface of poultry meat. However, subjection to washing and heat treatment may result in a loss of fat content (Pereira et al., 1976). Piette et al. indicated that high temperature (80°C) could effectively be used to extract fat from chicken skin (Piette et al., 2001). According to the methods adopted by the manufacturers of ready‐to‐eat ginseng chicken soup, the raw chicken is cooked before retorting to reduce the fat content and to improve the quality characteristics of their products (Triyannanto & Lee, 2015). In the study conducted by Triyannanto and Lee (2015), the fat content of ginseng chicken soup obtained by adding a washing step to the standard methodology was the lowest in three different treatments (addition of emulsifier; addition of emulsifier/precooking; or addition of emulsifier/precooking/washing). Fat in conventional food products is a source and carrier of flavor as well as a flavor enhancer. Several unsaturated fatty acids in fat have been proven to intensify the flavor of chicken (Jayasena et al., 2015). Among them, hexanal and 2,4‐decadienal, the most abundant aldehydes in chicken, are derived from the oxidative decomposition of linoleic acid (Shi & Ho, 1994). Arachidonic acid contributes to the umami taste of chicken (Jung et al., 2014; Kiyohara et al., 2011). Docosahexaenoic acid increases the sweetness and umami characteristics while inhibiting the sour and bitter tastes (Koriyama et al., 2002). Oleic acid reportedly increases taste panel scores for meat flavor (Wood & Enser, 1997). Altogether, the loss of fat directly leads to the reduction of flavor‐related fatty acids, which affects the flavor characteristics of chicken/chicken soup.

Postmortem aging is a necessary process for muscle to meat transformation, which is conducive to improving meat quality (Nishimura et al., 1988). Postmortem aging of chicken is another crucial factor affecting the taste of chicken soup. Nishimura et al. (1988) reported that chicken flavor was more pleasant at 8 hr than immediately after slaughter. In the process of postmortem aging of chicken, the glutamic acid level increases rapidly owing to the high activity of aminopeptidase and its hydrolytic activity toward glutamic acid‐β‐naphthylamide (Glu‐NA) (Nishimura et al., 1988). Considerable increases in alanine, serine, glutamic acid, and leucine could be obtained by postmortem aging of chicken breast muscle for 6 days at 4°C. Studies also revealed a marked increase in 5′‐IMP and 5′‐GMP content during postmortem aging of chiller, which results in a significant increase in the intensity of the savory, brothy taste of chicken (Nishimura et al., 1988; Tikk et al., 2006).

#### 4.3.2 Cooking

Cooking is a key factor affecting the flavor of chicken soup (Al‐Khalifa & Dawood, 1993). During stewing, water‐soluble taste substances produced in chicken and during heat treatment migrate to water, thereby resulting in a delicious taste. The ratio of meat to water, heating temperature and time, cooking method, and seasoning are important parameters affecting the quality of chicken soup. Studies have shown that higher meat to water ratio aided the extraction of the taste compounds from chicken. Furthermore, the contents of 5′‐IMP, 5′‐GMP, and lactic acid were found to increase (Chen et al., 2007). Prolongation of the stewing time also aided the extraction of the taste substances from chicken. Chen et al. (2007) revealed that the lactic acid content in chicken soup increased significantly when stewing time was extended. Furthermore, the 5′‐GMP and 5′‐IMP contents were found to reach the maximum level after performing heating for 2 hr. Cooking temperature has a significant effect on the hydrolysis of animal protein. Previous studies have shown that thermal treatment increased the activity of the meat enzymes up to a certain temperature (75°C). However, at higher temperatures, the proteolytic activity decreased, and Maillard and Strecker degradation reactions were potentiated (Zhang et al., 2013). The active taste compounds in the final soup product directly help determine the sensory quality of the soup. Zhang et al. (2013) studied the effect of cooking temperature on sensory characteristics and protein hydrolysates of crucian carp soup. Contents of total peptides, total free amino acids, and umami amino acids were found to be the highest at 85°C. Therefore, 85°C was considered as the best cooking temperature for improving the flavor and nutritional value of crucian carp soup (Zhang et al., 2013). In the study reported by Pérez‐Palacios et al. (2017), the hydrolysis of protein and the diffusion of amino acids occurred significantly in broilers when chicken soup was cooked at a controlled temperature of 85–103°C for 3, 4, and 5 hr; however, Strecker degradation and Maillard reaction were not significantly noted under these conditions. Therefore, compared to the values at 85°C, the values of amino acids, nucleotides, and equivalent umami were higher at 103°C (Pérez‐Palacios et al., 2017). Seasonings are often used to increase the acceptability of chicken soup. As early as 1992, monosodium glutamate (MSG) and sodium chloride (NaCl) concentrations had been proved to have a significant effect on the hedonic score of chicken soup (*p* < .05) (Chi & Chen, 1992). Chi and Chen (1992) reported that spiced chicken soup (adding 0.125% granulated onion, 0.0125% garlic powder, 0.0125% ground white pepper, and 0.0125% whole celery seed) had a higher maximum hedonic score (7.81, near to like very much) than nonspiced chicken soup (7.28, near to like moderately), which indicated that the spice contributes to the hedonic score of chicken soup (Chi & Chen, 1992). In addition, the addition of scallop and celery components were also contributed to the sweetness and umami taste of chicken soup (Kurobayashi et al., 2008; Yoneda et al., 2005).

### 4.4 Storage

With the degradation of ATP and protein, several taste compounds are produced during the postmortem storage of chicken, which significantly improves the taste of chicken soup. Furthermore, most free and combined amino acids, ammonia, inosine, and hypoxanthine increase during storage. Studies have shown that soup prepared from the chicken muscle stored for 7 or 8 days at 0°C exhibited the highest taste intensity based on sensory evaluation (Sen & Endo, 1990). The chicken soup as a system is complicated. In fact, the chemical and biochemical changes caused by microorganisms during the storage process lead to the deterioration of the sensory properties of chicken soup. Studies have shown that chicken whey soup has a shelf life of 6 days when refrigerated (Chidanandaiah et al., 2002). In the study reported by Gadekar et al. (2009), there was a significant (*p* <.05) interaction between the refrigerated storage period and treatments conducted for flavor and overall palatability of soup.

Generally, lower storage temperature and better packaging materials can help maintain the taste of chicken soup. Low temperature could inhibit the growth of microorganisms and enzyme activity, thereby extending the shelf life of chicken soup (Sivertsvik et al., 2003). Polypropylene/active zein bags (10% Lauroyl‐l‐arginine ethyl ester monohydrochloride [LAE]) were developed as the packaging material for chicken soup to control the growth of foodborne pathogens (Kashiri et al., 2019). In the study reported by Kashiri et al. (2019), the antimicrobial properties of the PP/LAE glycerol‐plasticized zein bags caused a reduction of 3.21 and 3.07 log against *Listeria monocytogenes* and *Escherichia coli*, respectively, in chicken soup after 10 days of storage (4°C). Thermal processing and the adoption of better sanitary procedures during soup processing could significantly inhibit the growth of coliforms, thereby prolonging the storage time of chicken soup (Gadekar et al., 2009).

## 5 CONCLUSION

The taste of chicken soup is a result of the interaction of several taste compounds. Identification of the taste substances in chicken soup has remarkable significance. To date, more than 91 taste‐active compounds have been reported in chicken/chicken soup. Among these compounds, 5′‐IMP is the component responsible for the umami taste of chicken meat. Apart from 5′‐IMP, amino acids and their derivatives, organic acids, and peptides also strongly affect the taste quality of chicken soup. Liquid chromatography–mass spectrometry is the most commonly used method for conducting qualitative and quantitative analysis of the taste components in chicken soup. The combination of human tongue and the innovative electronic tongue system can not only enable the identification of the taste attributes and strength of chicken soup, but also facilitate the quantification of less remarkable differences among samples. Chicken soups prepared with the same breed of chicken have different tastes and qualities due to differences in preslaughter factors, processing, and storage. Washing can significantly reduce the fat content in chicken soup and affect its flavor characteristics. Aging and prolonged duration of stewing are effective methods for improving the taste of chicken soup. The production of off‐flavor substances and the loss of characteristic taste substances are the main reasons for the deterioration of the taste quality of chicken soup. Storage time and storage conditions were found to significantly affect the biochemical reaction of chicken soup, thereby affecting its taste during storage. More than 300 compounds have been reported in chicken/chicken soup. Furthermore, its taste characteristics are attributed to several taste compounds. Different proportions of these characteristic taste substances may lead to different perception (taste intensity, attributes, and so on) results. In general, the relationship between taste‐active compounds and the taste of chicken soup has not been well established. Therefore, characteristic taste‐active compounds and their relationship with the sensory attribute must be investigated in future studies.

## CONFLICTS OF INTEREST

The authors declare that there are no conflicts of interest.

## AUTHOR CONTRIBUTIONS

**Lili Zhang:** Data curation (equal); Investigation (equal); Methodology (equal); Writing‐original draft (equal); Writing‐review & editing (equal). **Zhilin Hao:** Data curation (equal); Methodology (equal); Visualization (equal); Writing‐review & editing (equal). **Chao Zhao:** Methodology (equal); Writing‐review & editing (equal). **Yuyu Zhang:** Methodology (equal); Resources (equal); Writing‐review & editing (equal). **Jian Li:** Methodology (equal); Writing‐review & editing (equal). **Baoguo Sun:** Resources (equal). **Yizhuang Tang:** Writing‐review & editing (equal). **Meixiang Yao:** Writing‐review & editing (equal).
